# Nitrous Oxide and Oxygen in Combination with Ether or C. E. Mixture for Nose and Throat Operations

**Published:** 1919

**Authors:** 


***Nitrous Oxide and Oxygen in Combination with Ether or C. E. Mixture for Nose and Throat Operations.*** By Capt. Edmund G. Boyle. *The British Medical Journal,* December 21, 1918.

Having had a large experience in civilian surgery of the nose and throat, the author tells of the technic employed in anesthesia by the use of nitrous oxide-oxygen and ether.

Three points are specially emphasized :

(1) The exceedingly small amount or C. E. mixture that is required.

(2) The enormous improvement in the patient's well-being.

(3) The excellence of the anesthesia.

Technic : A hypodermic of morphine and atropine half an hour before operation, varied according to the condition of the patient, occasionally adding scopolamine. Largest military dose 1/4 gr. morphine, 1/100 gr. atropine, 1/100 gr. scopolamine; for women 1/6 gr. morphine, atropine 1/100. Patient is anesthetized with nitrous oxide and oxygen by the rebreathing method. Just before anesthesia is reached the nitrous oxide and oxygen is allowed to run through the ether or C. E. mixture until the required depth is obtained; the face-piece is then removed and anesthesia maintained by running the combination through the nose or into the mouth. The patient's condition will determine whether the mixture must be given throughout operation; usually it is unnecessary.

If the air passages are not unduly obstructed, this anesthesia will meet the requirements of most operators. The patient should be pink, and cyanosis or pallor is indicative of bad administration. This can be corrected by oxygen.

In many cases only one drachm of C. E. mixture was necessary.

The method described is simple, but requires thorough acquaintance with the administration of nitrous oxide and oxygen with rebreathing. It is useful in tonsillectomy, resection of septum, bronchoscopies and esophagoscopies, making a quiet, easy anesthesia with quick return to comfort on the part of the patient. Anesthesia is adequate and can be prolonged to suit the need of the operator, and the patient is not nauseated afterward.

Observations : Complete relaxation of jaw and soft palate is obtained; swallowing and cough reflexes are easily abolished; bleeding is less; there is rapid recovery to complete consciousness.

In the administration of more than three thousand anesthetics *there have been no deaths.*

